# Bio-Based Polyurethane and Its Composites towards High Damping Properties

**DOI:** 10.3390/ijms23126618

**Published:** 2022-06-14

**Authors:** Shikai Hu, Yaowen Wu, Guoqing Fu, Tao Shou, Mengyao Zhai, Dexian Yin, Xiuying Zhao

**Affiliations:** 1 State Key Laboratory of Organic-Inorganic Composites, Beijing University of Chemical Technology, Beijing 100029, China; skhu@mail.buct.edu.cn (S.H.); w18813077124@163.com (Y.W.); fgq098152@163.com (G.F.); tshou14@163.com (T.S.); abc18336125632@163.com (M.Z.); yindexian96@163.com (D.Y.); 2 Beijing Engineering Research Center of Advanced Elastomers, Beijing University of Chemical Technology, Beijing 100029, China; 3 Engineering Research Center of Elastomer Materials on Energy Conservation and Resources, Ministry of Education, Beijing 100029, China

**Keywords:** composites, PLA-based polyols, polyurethane, AO-80, damping property

## Abstract

The operation of mechanical equipment inevitably generates vibrations and noise, which are harmful to not only the human body but also to the equipment in use. Damping materials, which can convert mechanical energy into thermal energy, possess excellent damping properties in the glass transition region and can alleviate the problems caused by vibration and noise. However, these materials mainly rely on petroleum-based resources, and their glass transition temperatures (*T_g_*) are lower than room temperature. Therefore, bio-based materials with high damping properties at room temperature must be designed for sustainable development. Herein, we demonstrate the fabrication of bio-based millable polyurethane (BMPU)/hindered phenol composites that could overcome the challenges of sustainable development and exhibit high damping properties at room temperature. BMPUs with a high *T_g_* were prepared from modified poly (lactic acid)-based polyols, the unsaturated chain extender trimethylolpropane diallylether, and 4,4′-diphenylmethane diisocyanate, and 3,9-Bis-{1,1-dimethyl-2[β-(3-tert-butyl-4-hydroxy-5-methylphenyl-)propionyloxy]ethyl}-2,4,8,10-tetraoxaspiro [5,5]-undecane (AO-80) was added to prepare BMPU/AO-80 composites. Finally, the properties of the BMPUs and BMPU/AO-80 composites were systematically evaluated. After adding 30 phr of AO-80, the Tg and maximum loss factor (tan δ_max_) of BMPU/AO-80 composites increased from 7.8 °C to 13.5 °C and from 1.4 to 2.0, respectively. The tan δ_max_ showed an improvement of 43%. Compared with other polyurethanes, the prepared BMPU/AO-80 composites exhibited higher damping properties at room temperature. This study proposes a new strategy to reduce society’s current dependence on fossil resources and design materials featuring high damping properties from sustainable raw materials.

## 1. Introduction

All types of mechanical equipment cause vibrations and different levels of noise during their operation [1,2]. These vibrations and noises are harmful to not only the human body but also to the equipment in use [3,4]. Thus, damping materials that can transform mechanical vibration energy into heat energy and dissipate it must be developed [5,6]. Rubber is the most widely used polymer-damping material owing to its viscoelastic properties [7,8]. However, the raw materials used to produce synthetic rubber are derived from fossil fuels [9]. According to the statistical data of BP energy published in 2021, oil, natural gas, and coal can be used for 53.5, 40.8, and 139 years, respectively [10]. Biomass is the most abundant renewable resource in the world, ranking fourth in terms of total energy consumption after coal, oil, and natural gas [10,11]. According to the 2021 global bioenergy statistical report data released by the world bioenergy association, the total global energy consumption in 2019 reached 379 EJ, while the biomass supply was 56.9 EJ, accounting for 15% of the total global energy consumption [12]. Thus, the development of bio-based elastomers, instead of traditional petroleum-based ones, is an important means to reduce resource consumption. As a bio-based polymer, poly(lactic acid) (PLA) is an excellent low-carbon material that meets environmental requirements. The most remarkable characteristic of PLA is that its raw materials come from renewable plant resources; they can also be recycled and do not depend on petroleum resources [13,14]. The synthesis of PLA from the fermentation of plant raw materials has been previously reported [15,16,17]. Abandoned PLA under the condition of the compost can be degraded into carbon dioxide and water, and degradation products by the photosynthesis of plants can also form materials such as starch, and the raw materials lactic acid fermented into PLA [18,19]. However, PLA shows poor toughness [20,21,22].

Researchers have sought to address the problems presented by PLA and improve its comprehensive properties by chemical modification and physical blending [23,24]. The molecular structure of polyurethane is highly tailorable, and its glass transition temperature (*T_g_*) and other properties can be controlled by adjusting the proportion of soft and hard segments, as well as the types and molecular weights of the soft segments [25,26,27]. PLA has also been introduced into polyurethane molecules to modify the latter. Previous polyurethanes based on PLA are plastic at room temperature because of the high molecular weight of their soft segments and, thus, cannot be used as elastomers [28,29]. A series of PLA-based TPUs were prepared using PLA as soft segments; the *T_g_* values of these materials were close to room temperature, and their damping performance was enhanced compared with other polyurethanes at room temperature [30,31]. However, the tensile strength of the samples was low, and they appeared to yield under high hard segment contents and show permanent deformation owing to the absence of chemical cross-linking. Millable polyurethanes can be processed like rubber, and their hard segments can be cross-linked by introducing an unsaturated chain extender. Materials with side groups are beneficial for improving the property of damping materials [32,33]. Composites can exploit the advantages of each component [34,35,36,37,38]. Thus, organic polar small molecules, such as hindered phenols and amines, are widely used to improve the *T_g_* and loss factor of polar substrates via the formation of hydrogen bonds between the large hydroxyl, ester, ether, and amine groups of these molecules and polar groups in the matrix [39,40,41,42].

In this study, a series of bio-based millable polyurethanes (BMPUs) were synthesized from modified PLA-based polyols, 4,4′-diphenylmethane diisocyanate (MDI), and unsaturated chain extender trimethylolpropane diallylether (TME). 3,9-Bis-{1,1-dimethyl-2[β-(3-tert-butyl-4-hydroxy-5-methylphenyl-) propionyloxy] ethyl}-2,4,8,10-tetraoxaspiro [5,5]-undecane (AO-80) was then added to the BMPUs to prepare BMPU/AO-80 composites. The properties of the BMPUs and BMPU/AO-80 composites were systematically studied. The results showed that the BMPUs and BMPU/AO-80 composites demonstrate excellent comprehensive properties, including high damping and tensile strength. The tan δ_max_ of the composites increased from 1.4 to 2.0, an improvement of 43%, after adding 30 phr of AO-80. This study provides a sustainable route for developing new materials with high damping properties and tensile strength at room temperature.

## 2. Results and Discussion

### 2.1. Preparation of BMPUs and BMPU/AO-80 Composites

**Figure 1:**
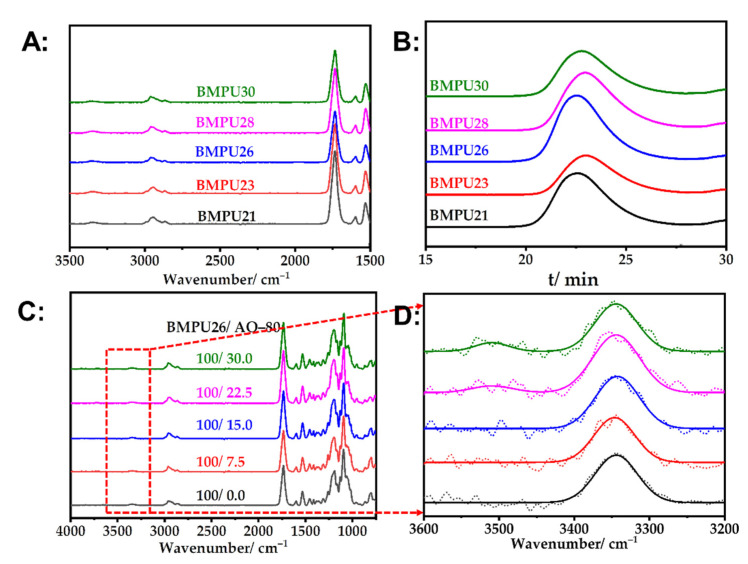
(**A**) FTIR spectra and (**B**) GPC curves of the BMPUs. (**C**) FTIR spectra of the BMPU/AO-80 composites. (**D**) Original and multipeak fitted FTIR spectra of the BMPU/AO-80 composites in the 3280–3610 cm^−1^ region.

The spectra of BMPUs with different hard segment contents are shown in Figure 1A. The *v*(NH) peak appeared at 3345 cm^–1,^ but no characteristic peak of the –NCO group was detected at 2250 cm^−1^. This result indicates that MDI had completely reacted with the PLA-based polyols and trimethylolpropane diallylether and that the BMPUs were successfully synthesized. The GPC curves of the BMPUs are shown in Figure 1B. The average molecular weight and molecular weight distribution index of the BMPUs were 2.4–3.2 × 10^4^ and 2.0, respectively. The FTIR spectra of the BMPU/AO-80 composites are shown in Figure 1C. To study the effect of AO-80 on the BMPU/AO-80 composites, we used infrared multipeak fitting to fit the spectra of these composites measured over the wavelength range of 3280–3610 cm^−1^. The spectra of the BMPU/AO-80 composites did not exhibit the characteristic peak of the free hydroxyl group of AO-80 at 3600 cm^–1^; instead, this peak was observed between 3590 and 3500 cm^–1^ and appeared to red-shift with increasing AO-80 content. This finding confirms that the hydroxyl group of AO-80 forms hydrogen bonds with the polar groups of the BMPU and that these bonds are enhanced with increasing AO-80 content [39,42]. This effect may be attributed to the formation of intermolecular hydrogen bonds between the hydroxyl groups of AO-80 and t the ester and carbamate groups of the BMPU matrix.

### 2.2. Curing of the BMPUs and BMPU/AO-80 Composites

**Figure 2:**
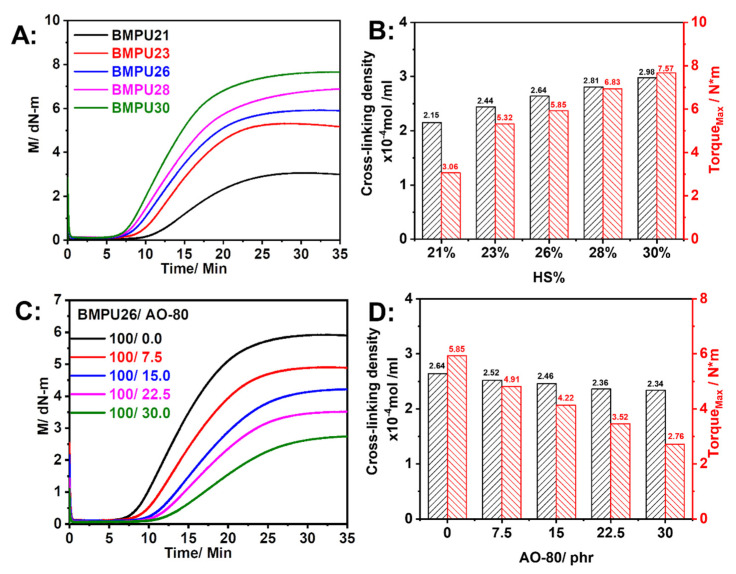
(**A**) Vulcanisation curves and (**B**) cross-linking density and torque_Max_ of BMPUs. (**C**) Vulcanization curves, (**D**) cross-linking density, and torque_Max_ of BMPU/AO-80 composites.

The vulcanization curves and cross-linking density of BMPUs with different hard segment contents are shown in Figure 2A,B, respectively. The torque difference (ΔM) and cross-linking density of the BMPUs increased with increasing hard segment content, which increases the number of unsaturated double bonds in the polyurethane molecular chains and, in turn, the number of chemical cross-linking points. Therefore, the cross-linking density and ΔM of the BMPUs increased [43]. The vulcanization curves and cross-linking densities of BMPU/AO-80 composites with different AO-80 contents are shown in Figure 2C,D, respectively. The ΔM and cross-linking density of the BMPU/AO-80 composites decreased with increasing AO-80 content because AO-80 melts at high temperatures and acts as a plasticizer, resulting in a decrease in the maximum torque of the composites. In addition, vulcanization of the BMPU/AO-80 composites involved free-radical crosslinking; however, the free-radical scavenging ability of AO-80 affected this cross-linking process.

**Figure 3:**
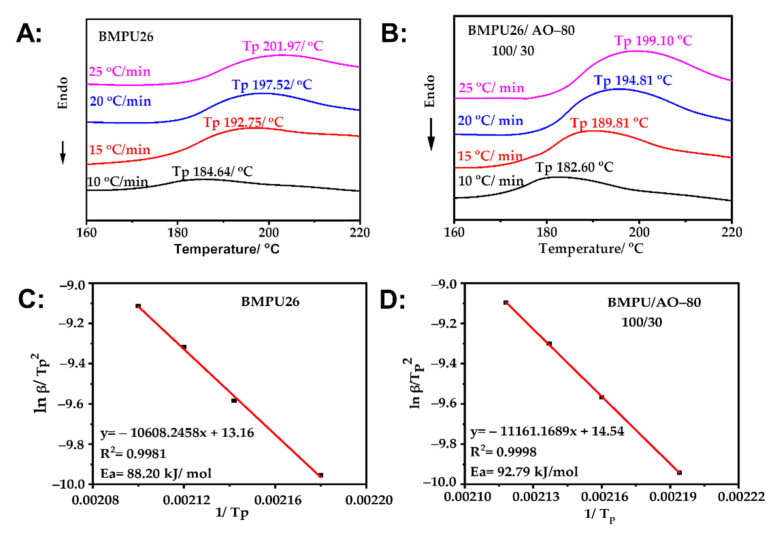
(**A**) DSC curves of BMPU26 at different heating rates. (**B**) DSC curves of BMPU26/AO-80(100/30) at different heating rates. (**C**) Dependence of ln(βTp^–2^) on Tp^–1^ for BMPU26. (**D**) Dependence of ln(βTp^–2^) on Tp^–1^ for BMPU26/AO-80(100/30).

The DSC curves of BMPU26 and BMPU26/AO-80(100/30) during curing at different heating rates are shown in Figure 3A,B. The characteristic exothermic temperatures increased with increasing heating rates. The original Kissinger method was used to calculate the apparent activation energy (Ea) of the specimens [44,45]. The kinetic parameters calculated using Equation (1) are listed in Table 1, where *β* is the heating rate, *R* is the gas constant (8.314 J/(mol∙K)), and C is a constant. Thus, a straight line can be obtained from plotting lnβTp^–2^ versus Tp^–1^ (Figure 3C,D), and the slope of this line is equal to—EaR^−1^ from which Ea could be obtained. The Ea values of BMPU26 and BMPU26/AO-80(100/30) were 88.20 and 92.79 kJ/mol, respectively. The Ea of BMPU26 was larger than that of BMPU26/AO-80 (100/30), which suggests that AO-80 has a negative effect on vulcanization, consistent with the vulcanization and cross-linking density results.

ln(βT_p_^−2^) = C − (EaR^−1^T_p_^−1^) (1)

### 2.3. DSC and XRD Analyses of the BMPUs and BMPU/AO-80 Composites

**Figure 4:**
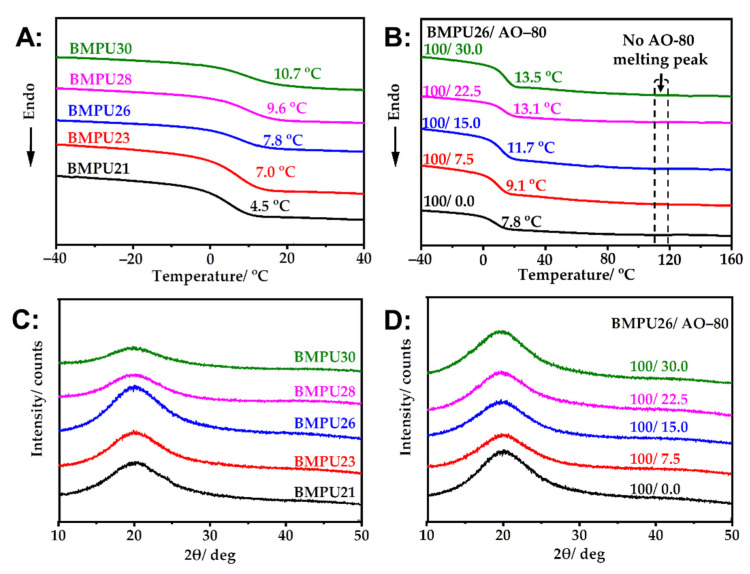
DSC curves of the (**A**) BMPUs and (**B**) BMPU/AO-80 composites. XRD curves of the (**C**) BMPUs and (**D**) BMPU/AO-80 composites.

As shown in Figure 4A, the *T_g_* of the BMPUs increased with increasing hard segment content, which increases the cross-linking density of the specimens (see Section 3.2) and limits the motion of soft segments. As the hard segment content increases in the samples, higher energy is needed to achieve chain segment motion; thus, the *T_g_* of the BMPUs increases. As shown in Figure 4B, no crystal peak of AO-80 was observed in the BMPU/AO-80 composite at 119 °C [46], indicating that AO-80 formed a good dispersion in the BMPU matrix. The *T_g_* of the BMPU/AO-80 composites further increased with increasing AO-80 dosage because AO-80 can produce hydrogen-bonding interactions with polar groups in the BMPU matrix [39,42] (see Section 3.1), which limits the movement of soft segments in the composites. The XRD curves of the BMPUs and BMPU/AO-80 composites are shown in Figure 4C,D, respectively. No characteristic crystal peaks were observed, which is consistent with the DSC results.

### 2.4. Static and Dynamic Mechanical Analyses of BMPUs and BMPU/AO-80 Composites

**Figure 5:**
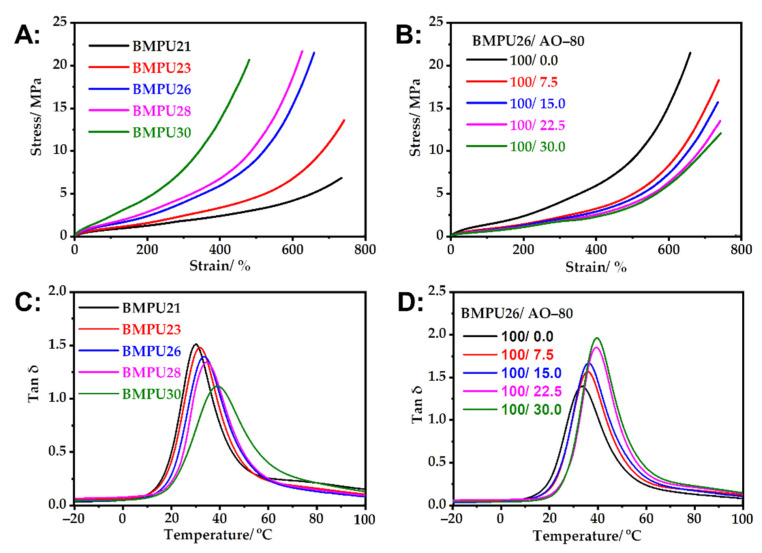
Stress–strain curves of the (**A**) BMPUs and (**B**) BMPU/AO-80 composites. DMA curves of the (**C**) BMPUs and (**D**) BMPU/AO-80 composites.

As shown in Figure 5A and Table S1, the tensile strength of the BMPUs increased with increasing hard segment content, whereas their elongation at break decreased. This finding may be attributed to the increase in the chemical cross-linking density of the samples. Increased hydrogen-bonding interactions between hard segments also increase physical cross-linking with increasing hard segment content. Thus, the samples can resist greater stress. Moreover, the fracture and recombination of the hydrogen bonds of the BMPUs lead to higher energy requirements to maintain their tensile properties. A higher cross-linking density leads to greater restrictions on the movement of molecular chains, resulting in a decrease in the elongation at break. A large number of hydrogen-bonding interactions can delay the process of chemical-bond fracturing in the BMPU matrix. The tensile strength of the BMPU/AO-80 composites decreased slightly, whereas their elongation at break increased slightly with increasing AO-80 content (Figure 5B and Table S1). This is because the free radical scavenging ability of AO-80 affected this cross-linking process, thereby destroying the cross-linking network. As a result, the cross-linking density of the materials decreased (see Section 3.2).

The *T_g_* and the storage modulus in the rubber state increased with increasing hard segment content (Figure 5C and Figure S1A) owing to the increase in cross-linking density and reinforcing effect of the hard segments. With increasing hard segment content, the movement of the soft segments of the BMPUs is restricted, the friction between soft segments decreases, and the energy dissipation ability of the samples decreases. However, with increasing hard segment content, the relative content of the soft segment of BMPU decreases, resulting in a reduction in the maximum loss factor (tan δ_max_), as shown in Figure 5C and Table S2. A hard segment with a large average molecular size has a significant reinforcing effect on the BMPUs, such that the storage modulus of the samples in the rubber state increases significantly. The *T_g_* of the BMPU/AO-80 composites increased with increasing AO-80 content, which is consistent with the DSC results. The tan δ_max_ of the BMPU/AO-80 composites also increased (Figure 5D and Table S2). AO-80 can form strong hydrogen bonds with polar groups in the BMPU matrix [39,42] (see Section 3.1). Hydrogen bonds are intermolecular forces that are stronger and consume more energy than intermolecular friction during motion.

**Figure 6:**
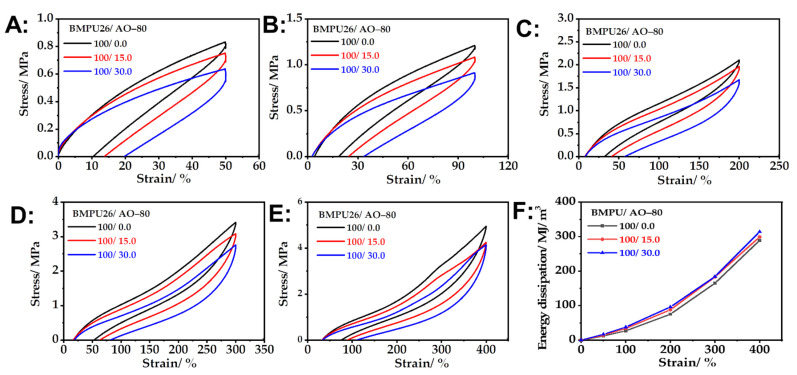
Tensile loading–unloading curves of the BMPU/AO-80 composites at strains of (**A**) 50%, (**B**) 100%, (**C**) 200%, (**D**) 300%, and (**E**) 400%. (**F**) Energy dissipation of the BMPU/AO-80 composites.

The tensile recovery of the BMPU/AO-80 composites was studied at strains of 50%, 100%, 200%, 300%, and 400%. The experimental results are shown in Figure 6. The lag-ring area increased with the increasing amount of AO-80. The area of the closed curve in the tensile cycle represents the energy loss of the material during the tensile cycle [47]. Because of the unique viscoelasticity of the BMPU/AO-80 composites, their strain lagged behind their stress, resulting in a large amount of friction in the macromolecular chains of the materials. When the BMPU/AO-80 composites were subjected to an external tensile force, hydrogen bond fracture and recombination occurred, resulting in the consumption of large amounts of energy and increased energy dissipation. This finding indicates that the damping properties of the BMPU/AO-80 composites increase with increasing AO-80 content, which is consistent with the DMA results.

### 2.5. Comparison of the Overall Performance of BMPU/AO-80 Composites with Other Polyurethanes

**Figure 7:**
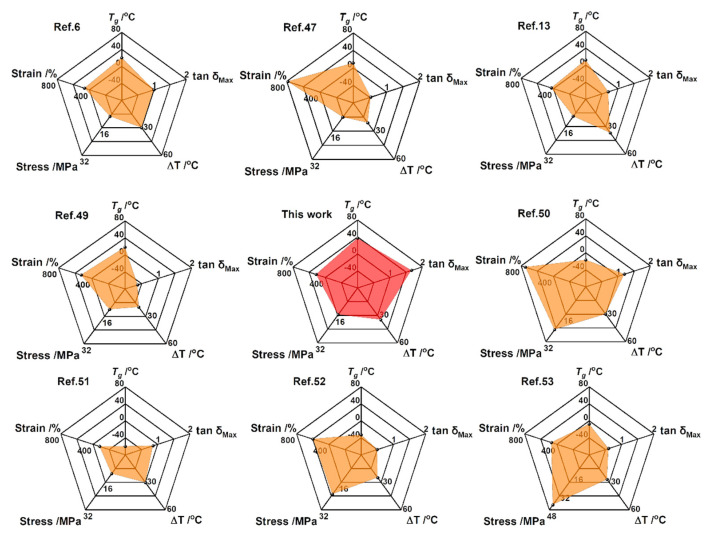
Damping and mechanical properties of different polyurethanes.

*T_g_*, tan δ_Max_, effective damping temperature range (temperature range of tan δ > 0.3), tensile strength, and elongation at break are important parameters for damping materials. The damping properties of the prepared BMPU/AO-80 composites outperformed those of most other polyurethanes, and their effective damping temperature range covered the room temperature range. These findings indicate that the fabricated composites may represent a new type of room-temperature damping material with excellent comprehensive properties. The results further showed that the *T_g_*, tan δ_Max_, effective damping temperature range, tensile strength, and elongation at break of BMPU/AO-80(100/15) were 37 °C, 1.7, 35.9 °C, 15.6 MPa, and 733%, respectively. To highlight the comprehensive properties of the BMPU/AO-80 composites, we selected a series of polyurethane elastomers and compared their properties with those of BMPU/AO-80(100/15) [6,48,49,50,51,52,53,54]; the results are shown in Figure 7. Among the polyurethane-based damping materials investigated, BMPU/AO-80(100/15) exhibited the best overall properties.

## 3. Material and Methods

### 3.1. Materials

Modified PLA-based polyols (*M_n_* = 2000 g/mol) were purchased from eSUN Industrial Co., Ltd. (Shenzhen, China). MDI and TME were purchased from Acros. Hindered phenol AO-80 was purchased from Asahi Denka Co., Ltd. (Tokyo, Japan). Stearic acid was purchased from Fengyi Oil Technology Co., Ltd. (Shanghai, China). Dibenzothia-zole disulfide and 2-Mercaptobenzothiazole were purchased from Kemai Chemical Co., Ltd. (Tianjin, China). Sulfur was purchased from Deli Chemical Co., Ltd. (Guangzhou, China). The raw materials were used as received without any treatment.

### 3.2. Fabrication of BMPUs and BMPU/AO-80 Composites

**Scheme 1:**
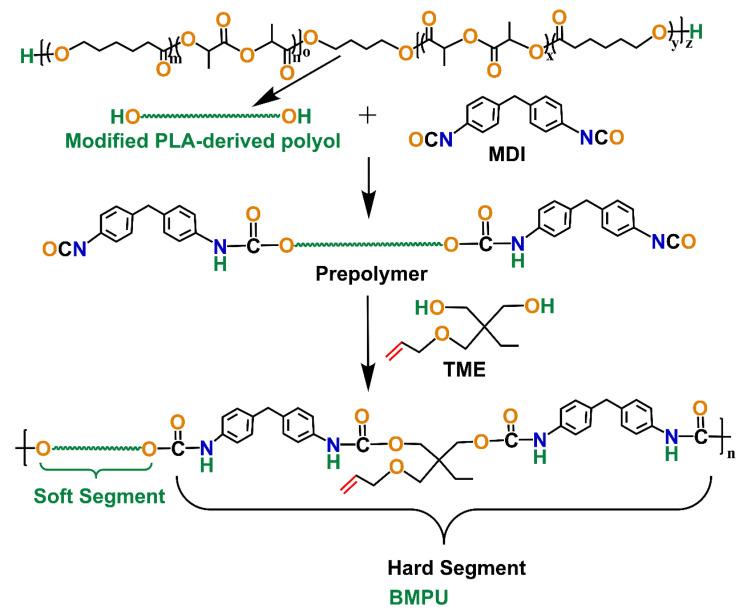
Synthetic route to the BMPUs.

Modified PLA-based polyols, MDI, and TME, were used to prepare the BMPUs. The synthetic route to the BMPUs is shown in Scheme 1. The ratio of the –OH groups in the modified PLA-based polyols and unsaturated chain extender to the –NCO groups in MDI was equal. The BMPUs were prepared as follows. First, the modified PLA-based polyols were dehydrated at 115 °C under a vacuum for 2 h. Next, MDI was added to the modified PLA-based polyols, and the mixture was allowed to react for 10 min at 80 °C to obtain the prepolymer. Finally, the prepolymer was mixed with TME and poured into a mold at 100 °C for 12 h. An internal mixer was used to uniformly mix the sulfur, other vulcanizing agents, and AO-80. The detailed compositions of the samples are shown in Table 2. The samples were designated as BMPU-XX, where XX represents the hard segment content.

### 3.3. Characterization

The Fourier transform infrared (FTIR) spectra of the BMPUs and BMPU/AO-80 composites were measured in attenuated total reflection mode on a Tensor 27 FTIR spectrophotometer with a resolution of 4 cm^−1^ (Tensor 27, Bruker, Karlsruhe, Germany). The FTIR spectra of the BMPU/AO-80 composites at 3280–3610 cm^−1^ were fitted using multipeak fitting.

The molecular weights and molecular weight distributions of the BMPUs were determined by gel permeation chromatography (GPC1515, Waters, Milford, MA, USA) with tetrahydrofuran as the eluent (1.0 mL/min).

The cross-linking processes of the BMPUs and BMPU/AO-80 composites were investigated using a moving die rheometer (MR-C3, High Speed Rail Testing Instruments Co., Dongguan, China) at 150 °C to determine their hot-pressing time.

Nuclear magnetic resonance (NMR) spectroscopy (VTMR20-010V-1, Suzhou Niumag Corporation, Suzhou, China) was used to investigate the cross-linking density of the BMPUs and BMPU/AO-80 composites at a frequency of 15 MHz, magnetic induction intensity of 0.5 ± 0.05 T, and temperature of 90 °C.

A STARe system DSC instrument (Mettler-Toledo International, Inc., Zurich, Switzerland) was used to measure the DSC data of the BMPUs and BMPU/AO-80 composites. The samples were scanned from −100 to 200 °C at a rate of 10 °C/min under an N_2_ atmosphere (50 mL/min).

An X-ray diffractometer (2500VB2 + PC, Rigaku, Tokyo, Japan) was used to examine the crystallization properties of the samples. The scanning angles ranged from 5° to 90°.

The mechanical properties of the BMPUs and BMPU/AO-80 composites were measured at a rate of 50 mm/min using a tensile testing machine (Txet-port II, Zwick/Roell, Ulm, Germany). The cyclic tensile tests of the samples were conducted under the same conditions at strains of 50%, 100%, 200%, and 300%. The size of the dumbbell specimen was 25 mm × 4 mm × 1 mm.

A dynamic mechanical thermal analyzer (Q800, TA Instruments, Wilmington, DE, USA) was used to measure the dynamic mechanical properties of the BMPUs and BMPU/AO-80 composites in the temperature range of −80 to 100 °C at a rate of 3 °C/min with a measurement frequency of 10 Hz and an amplitude of ε = 0.1%.

## 4. Conclusions

In this study, BMPU was first synthesized using modified PLA-based polyols, MDI, and TME. BMPU/AO-80 composites were then prepared. The *T_g_* of the BMPU/AO-80 composites was controllably adjusted by modifying the amount of AO-80 added to the composites. Thus, the BMPU/AO-80 composites exhibited a *T_g_* near room temperature and excellent damping properties at this temperature. The tan δ_max_ of the BMPU decreased from 1.6 to 1.1 when the hard segment content increased from 21% to 30%. The tan δ_max_ of the composite was increased from 1.4 to 2.0 after adding 30 phr of AO-80. This study proposes a simple strategy for designing sustainable materials with high damping and controllable mechanical properties, which is also expected to inspire the related fields of engineering.

## Figures and Tables

**Table 1:** Temperatures of the main thermal events of the samples.

Samples	Heating Rate β (K/min)	Peak Temperature Tp (°C)	Peak Temperature Tp (K)	1/Tp	Ln(β/Tp^2^)
BMPU26	10	181.84	454.99	0.002198	−9.9380
	15	187.74	460.89	0.00217	−9.5583
	20	193.81	466.96	0.002142	−9.2968
	25	199.35	472.5	0.002116	−9.0972
BMPU26/AO-80(100/30)	10	182.60	455.75	0.002194	−9.9413
	15	189.81	462.96	0.002160	−9.5672
	20	198.81	467.96	0.002137	−9.3010
	25	199.10	472.25	0.002118	−9.0961

**Table 2:** Formulations of the BMPUs and BMPU/AO-80 composites.

Samples	Hard Segment Content (%)	BMPU(phr)	Stearic Acid (phr)	Dibenzothiazole Disulfide-ZnCl Complex (phr)	Dibenzothiazole Disulfide (phr)	2-Mercaptobenzothiazole (phr)	Sulfur (phr)	AO-80 (phr)
BMPU21	21	100	0.5	1	4	2	2	0
BMPU23	23	100	0.5	1	4	2	2	0
BMPU26	26	100	0.5	1	4	2	2	0
BMPU28	28	100	0.5	1	4	2	2	0
BMPU30	30	100	0.5	1	4	2	2	0
BMPU26/AO-80(100/7.5)	26	100	0.5	1	4	2	2	7.5
BMPU26/AO-80(100/15.0)	26	100	0.5	1	4	2	2	15
BMPU26/AO-80(100/22.5)	26	100	0.5	1	4	2	2	22.5
BMPU26/AO-80(100/30.0)	26	100	0.5	1	4	2	2	30

## Data Availability

The data that support the findings of this study are available on request from the corresponding author.

## Supplementary Materials

The following supporting information can be downloaded at: https://www.mdpi.com/article/10.3390/ijms23126618/s1.

## References

[1] Beniah G., Liu K., Heath W.H., Miller M.D., Scheidt K.A., Torkelson J.M. (2016). Novel thermoplastic polyhydroxyurethane elastomers as effective damping materials over broad temperature ranges. Eur. Polym. J..

[2] Zhang C., Chen Y., Li H., Liu H. (2018). Facile fabrication of polyurethane/epoxy IPNs filled graphene aerogel with improved damping, thermal and mechanical properties. RSC Adv..

[3] Zhou X., Yu D., Shao X., Zhang S., Wang S. (2016). Research and applications of viscoelastic vibration damping materials: A review. Compos. Struct..

[4] Zhou R., Gao W., Xia L., Wu H., Guo S. (2018). The study of damping property and mechanism of thermoplastic polyurethane/phenolic resin through a combined experiment and molecular dynamics simulation. J. Mater. Sci..

[5] Lv X., Huang Z., Huang C., Shi M., Gao G., Gao Q. (2016). Damping properties and the morphology analysis of the polyurethane/epoxy continuous gradient IPN materials. Compos. Part B Eng..

[6] Zhao X., Shou T., Liang R., Hu S., Yu P., Zhang L. (2020). Bio-based thermoplastic polyurethane derived from polylactic acid with high-damping performance. Ind. Crops Prod..

[7] Pochivalov K.V., Shilov A.N., Lebedeva T.N., Ilyasova A.N., Golovanov R.Y., Basko A.V., Kudryavtsev Y.V. (2021). Development of vibration damping materials based on butyl rubber: A study of the phase equilibrium, rheological, and dynamic properties of compositions. J. Appl. Polym. Sci..

[8] Zhu L., Chen X., Shi R., Zhang H., Han R., Cheng X., Zhou C. (2021). Tetraphenylphenyl-modified damping additives for silicone rubber: Experimental and molecular simulation investigation. Mater. Des..

[9] Lakhno Y.V. (2013). Development of the Russian market of synthetic rubber. Stud. Russ. Econ. Dev..

[10] BP (2021). BP Statistical Review of World Energy 2021.

[11] Vaclav S. (1983). Biomass Energies: Resources, Links, Constraints.

[12] World Bioenergy Association (2021). Global Bioenergy Statistics 2021.

[13] Sreekumar K., Bindhu B., Veluraja K. (2021). Perspectives of polylactic acid from structure to applications. Polym. Renew. Resour..

[14] Ilyas R.A., Zuhri M.Y.M., Aisyah H.A., Asyraf M.R.M., Hassan S.A., Zainudin E.S., Sapuan S.M., Sharma S., Bangar S.P., Jumaidin R. (2022). Natural Fiber-Reinforced Polylactic Acid, Polylactic Acid Blends and Their Composites for Advanced Applications. Polymers.

[15] Singhvi M.S., Zinjarde S.S., Gokhale D.V. (2019). Polylactic acid: Synthesis and biomedical applications. J. Appl. Microbiol..

[16] Seda T.A.R., Elvan A., Baki H. (2018). Soybean Oil Based Polylactic Acid Membranes: Synthesis and Degradation Characteristics. J. Polym. Environ..

[17] Van Wouwe P., Dusselier M., Vanleeuw E. (2016). Lactide Synthesis and Chirality Control for Polylactic acid Production. ChemSusChem.

[18] Zaaba N.F., Jaafar M. (2020). A review on degradation mechanisms of polylactic acid: Hydrolytic, photodegradative, microbial, and enzymatic degradation. Polym. Eng. Sci..

[19] Morozov A.G., Razborov D.A., Egiazaryan T.A., Baten’Kin M.A., Aleynik D.Y., Egorikhina M.N., Rubtsova Y.P., Charikova I.N., Chesnokov S.A., Fedushkin I.L. (2020). In Vitro Study of Degradation Behavior, Cytotoxicity, and Cell Adhesion of the Atactic Polylactic Acid for Biomedical Purposes. J. Polym. Environ..

[20] Dong J., Mao Z., Chen Z. (2022). Toughening, highly thermostable, and flame retardant polylactic acid enabled by polyphosphazene microsphere. J. Appl. Polym. Sci..

[21] Sun J., Jin Y., Wang B., Tian H., Kang K., Men S., Weng Y. (2021). High-toughening modification of polylactic acid by long-chain hyperbranched polymers. J. Appl. Polym. Sci..

[22] Zhang Q., Wang W., Gu X., Li H., Liu X., Sun J., Zhang S. (2019). Is there any way to simultaneously enhance both the flame retardancy and toughness of polylactic acid?. Polym. Compos..

[23] Martinez Villadiego K., Arias Tapia M.J., Useche J., Escobar Macías D. (2021). Thermoplastic Starch (TPS)/Polylactic Acid (PLA) Blending Methodologies: A Review. J. Polym. Environ..

[24] Liu Z., Lu H., Zhang H., Li L. (2021). Poly (vinyl alcohol)/polylactic acid blend film with enhanced processability, compatibility, and mechanical property fabricated via melt processing. J. Appl. Polym. Sci..

[25] Liu H. (2012). Handbook of Polyurethane Elastomers.

[26] Wen C.-H., Hsu S.-C., Hsu S.-H., Chang S.-W. (2019). Molecular Structures and Mechanisms of Waterborne Biodegradable Polyurethane Nanoparticles. Comput. Struct. Biotechnol. J..

[27] Zhang Q., Deng Y., Fu Z., Zhang H. (2019). Effects of the molecular structure on the vibration reduction and properties of hyperbranched waterborne polyurethane–acrylate for damping coatings. J. Appl. Polym. Sci..

[28] Oguz O., Candau N., Citak M.K., Cetin F.N., Seven S.A., Menceloglu Y.Z. (2019). A Sustainable Approach to Produce Stiff, Super-Tough, and Heat-Resistant Poly(lactic acid)-Based Green Materials(Article). ACS Sustain. Chem. Eng..

[29] Razali N.I.M., Ali F., Azmi A.S., Ismail T.N.M.T., Mirghani M.E.S., Omar M.F. (2021). Microwave-Assisted Synthesis of Polylactic Acid-Diol for Polyurethane as Biodegradable Packaging Material. IOP Conf. Ser. Mater. Sci. Eng..

[30] Jiang X., Qin R., Wang M., Xu M., Sheng Y., Lu X. (2021). Controllable wide temperature range and high damping polyurethane elastomer based on disulfide bond and dangling chain. Polym. Adv. Technol..

[31] Jiang X., Xu M., Wang M., Ma Y., Zhang W., Zhang Y., Rong H., Lu X. (2022). Preparation and molecular dynamics study of polyurethane damping elastomer containing dynamic disulfide bond and multiple hydrogen bond. Eur. Polym. J..

[32] Zhao Y., Shou T., Fu S., Qin X., Hu S., Zhao X., Zhang L. (2022). Controllable Design and Preparation of Hydroxyl-Terminated Solution-Polymerized Styrene Butadiene for Polyurethane Elastomers with High-Damping Properties. Macromol. Rapid Commun..

[33] Qi X., Xie F., Zhang J., Zhang L., Yue D. (2019). Bio-based cyclized Eucommia ulmoides gum elastomer for promising damping applications. RSC Adv..

[34] Díez-Pascual A.M. (2022). Biopolymer Composites: Synthesis, Properties, and Applications. Int. J. Mol. Sci..

[35] Hurtado A., Aljabali A.A.A., Mishra V., Tambuwala M.M., Serrano-Aroca Á. (2022). Alginate: Enhancement Strategies for Advanced Applications. Int. J. Mol. Sci..

[36] Qin Y., Li W., Liu D., Yuan M., Li L. (2017). Development of active packaging film made from poly (lactic acid) incorporated essential oil. Prog. Org. Coat..

[37] Sienkiewicz A., Czub P. (2020). Flame Retardancy of Biobased Composites—Research Development. Materials.

[38] Zafar K., Zia K.M., Alzhrani R.M., Almalki A.H., Alshehri S. (2022). Biocompatibility and Hemolytic Activity Studies of Synthesized Alginate-Based Polyurethanes. Polymers.

[39] Zhao X.-Y., Lu Y.-L., Xiao D.-L., Wu S.-Z., Zhang L.-Q. (2009). Thermoplastic Ternary Hybrids of Polyurethane, Hindered Phenol and Hindered Amine with Selective Two-Phase Dispersion. Macromol. Mater. Eng..

[40] Zhao X., Yang J., Zhao D., Lu Y., Wang W., Zhang L., Nishi T. (2015). Natural rubber/nitrile butadiene rubber/hindered phenol composites with high-damping properties. Int. J. Smart Nano Mater..

[41] Wang X., Chen X., Song M., Wang Q., Zheng W., Song H., Fan Z., Thu A.M. (2020). Effects of Hindered Phenol Organic Molecules on Enhancing Thermo-Oxidative Resistance and Damping Capacity for Nitrile Butadiene Rubber: Insights from Experiments and Molecular Simulation. Ind. Eng. Chem. Res..

[42] Xu K., Hu Q., Wang J., Zhou H., Chen J. (2019). Towards a Stable and High-Performance Hindered Phenol/Polymer-Based Damping Material Through Structure Optimization and Damping Mechanism Revelation. Polymers.

[43] Cui S., Li K., Shi Z., Zhang J., Liu J. (2021). Influence of hard segment content on the properties of millable polyurethane elastomer. Thermosetting Resin.

[44] Ortega A. (2008). A simple and precise linear integral method for isoconversional data. Thermochim. Acta.

[45] Rajeshwari P. (2016). Kinetic analysis of the non-isothermal degradation of high-density polyethylene filled with multi-wall carbon nanotubes. J. Therm. Anal..

[46] Xiang P., Xiao D., Zhao X., Lu Y., Zhang L. (2007). Microstructure and dynamic mechanical properties of hindered phenol AO-80/NBR/PVC crosslinking composites. J. Compos. Mater..

[47] Newman R.H., Battley M.A., Carpenter J.E.P., Le Guen M.J. (2012). Energy loss in a unidirectional flax-polyester composite subjected to multiple tensile load-unload cycles. J. Mater. Sci..

[48] Xiao S., Hossain M.M., Liu P., Wang H., Hu F., Sue H. (2017). Scratch behavior of model polyurethane elastomers containing different soft segment types. Mater. Des..

[49] Puszka A. (2018). Thermal and mechanical behavior of new transparent thermoplastic polyurethane elastomers derived from cycloaliphatic diisocyanate. Polymers.

[50] Dandeniyage L., Gunatillake P.A., Adhikari R., Bown M., Shanks R., Adhikari B. (2018). Development of high strength siloxane poly(urethane-urea) elastomers based on linked macrodiols for heart valve application. J. Biomed. Mater. Res. Part B Appl. Biomater..

[51] Xiao D., Zhao X., Feng Y., Xiang P., Zhang L., Wang W. (2010). The structure and dynamic properties of thermoplastic polyurethane elastomer/hindered phenol hybrids. J. Appl. Polym. Sci..

[52] Liu Y., Liu L., Liang Y. (2020). Relationship between structure and dynamic mechanical properties of thermoplastic polyurethane elastomer containing bi-soft segment. J. Appl. Polym. Sci..

[53] Kopczyńska P., Calvo-Correas T., Eceiza A., Datta J. (2016). Synthesis and characterisation of polyurethane elastomers with semi-products obtained from polyurethane recycling. Eur. Polym. J..

[54] Hu S., He S., Wang Y., Wu Y., Shou T., Yin D., Mu G., Zhao X., Gao Y., Liu J. (2022). Self-repairable, recyclable and heat-resistant polyurethane for high-performance automobile tires. Nano Energy.

