# Supplementary material for: Bio-Based Polyurethane and Its Composites towards High Damping Properties
Source: Int J Mol Sci. 2022 Jun 14;23(12):6618. doi: 10.3390/ijms23126618 (PMC9223548; doi:10.3390/ijms23126618)
Supplement: Supplementary file 1 [file ijms-23-06618-s001.zip › ijms-1762082-supplementary.pdf]

Table S1. Stress-strain data of BMPUs and BMPU/AO-80 composites.

Samples	Tensile strength/MPa	Elongation at break/%	Fracture toughness/ MJ/m^3
BMPU21	6.9±0.6	735±25	1879.71
BMPU23	13.6±0.5	741±23	3002.74
BMPU28	21.6±0.9	626±19	4076.07
BMPU30	20.6±0.7	480±30	4064.62
BMPU26	21.5 ± 0.8	660 ± 27	3513.76
BMPU26/AO-80(100/7.5)	18.3 ± 0.5	736 ± 20	3420.2
BMPU26/AO-80(100/15.0)	15.6 ± 0.7	733 ± 22	2987.07
BMPU26/AO-80(100/22.5)	13.5 ± 0.8	741 ± 19	2658.07
BMPU26/AO-80(100/30.0)	12.0 ± 0.7	743 ± 22	2485.34

Table S2. DMA results of BMPUs and BMPU/AO-80 composites.

Samples	T_g/°C	Tan δ_{max}	Tan δ >0.3		
			T1/°C	T2/°C	△T/°C
BMPU21	30	1.6	16.9	54.0	37.1
BMPU23	31	1.5	18.1	54.2	36.1
BMPU28	34	1.3	21.7	57.1	35.4
BMPU30	39	1.1	22.9	63.2	40.3
BMPU26	33	1.4	20.2	56.1	35.9
BMPU26/AO-80(100/7.5)	36	1.6	22.8	57.6	34.8
BMPU26/AO-80(100/15.0)	37	1.7	22.9	58.7	35.8
BMPU26/AO-80(100/22.5)	39	1.9	26.1	61.3	35.2
BMPU26/AO-80(100/30.0)	40	2.0	26.3	64.8	38.5

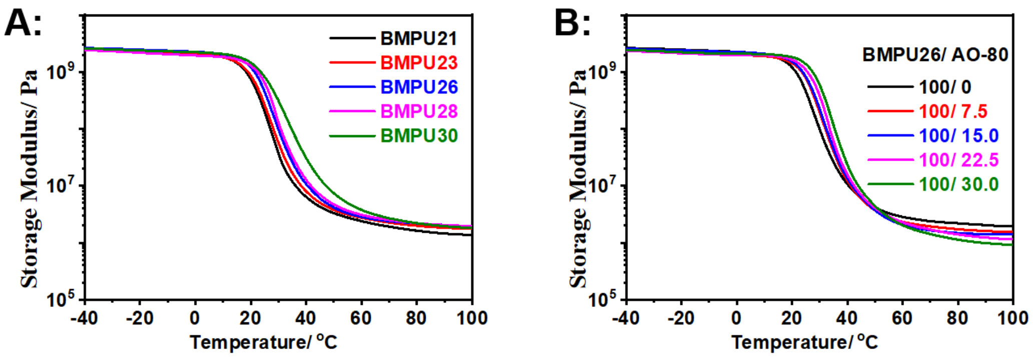

Figure S1. (**A**,**B**) Storage modulus of BMPUs and BMPUs/ AO-80 composites
